# *Hi-fun* among men who have sex with men in Bangkok: A scoping study exploring key informants’ perspectives on *hi-fun* contexts, harms and support strategies

**DOI:** 10.1371/journal.pgph.0002295

**Published:** 2023-08-25

**Authors:** T. Charles Witzel, Mookarpa Charoenyang, Adam Bourne, Thomas E. Guadamuz

**Affiliations:** 1 Department of Public Health, Environments and Society, London School of Hygiene and Tropical Medicine, London, United Kingdom; 2 Faculty of Social Sciences and Humanities, Center of Excellence in Research on Gender, Sexuality and Health, Mahidol University, Bangkok, Thailand; 3 Institute for Global Health, University College London, London, United Kingdom; 4 Australian Research Centre in Sex, Health and Society, La Trobe University, Melbourne, Australia; 5 Kirby Institute, University of New South Wales, Sydney, Australia; 6 John F. Kennedy School of Government, Harvard University, Cambridge, Massachusetts, United States of America; National University of Singapore, SINGAPORE

## Abstract

The use of specific drugs (e.g. methamphetamine, GHB/GBL and other stimulants) to enhance sex among men who have sex with men (MSM), is the focus of global public health concern because of links to social harms, poor mental and sexual health. Often called ‘chemsex’ in Western settings or ‘*hi-fun*’ in Southeast Asia, this type of sexualised drug use is increasingly visible in Thailand where the unique sociocultural and legislative environments shape sexual cultures and harms. This study aimed to develop an understanding of key informants’ perspectives on *hi-fun* contexts, harms and current responses in Bangkok. In-depth interviews were conducted with thirteen key informants from clinical, community, policy and development organisations. Four key informants had personal experience of *hi-fun*. Interviews covered *hi-fun* contexts, harms and support, were transcribed verbatim, translated to English (where necessary) and analysed using a thematic framework. MSM *hi-fun* ‘influencers’ shape norms and provide support online, primarily through Twitter. *Hi-fun* was linked to Westernisation and wealth; complex hierarchies emerged from asymmetries in social/financial capital. Police coercion towards MSM engaged in *hi-fun* was a concern. Given the nature of their funding, HIV/HCV/STI transmission was the most pressing focus for many organisations, however key informants were concerned especially about drug overdoses and mental health/well-being impacts. The political and economic context means funding for MSM health in Thailand focuses primarily on HIV prevention/treatment; restrictions on development aid constrain holistic *hi-fun* focused service development. Most *hi-fun* support was informally developed; successful strategies relied on partnership working and peer developed/delivered services, some of which were adapted from high-income settings. Despite substantial barriers, organisations developed services responding to the needs of MSM engaged in *hi-fun*. Given that many were informally developed or adapted from high-income settings, establishing a theoretical basis for further interventions that is grounded in this unique context is a priority.

## Introduction

Recent years have seen increased reporting of psychoactive substance use by men who have sex with men (MSM) in sexual contexts to enhance pleasure, longevity and arousal. Termed ‘*chemsex’* or ‘*party-n-play’* in Western settings or ‘*hi-fun*’ in Southeast Asia, this type of sexualised drug use (SDU) is facilitated by social media and hook-up apps [1]. Drugs typically associated are methamphetamine, ketamine, gamma hydroxybutyrate/gamma butyrolactone (GHB/GBL) and other stimulants [1]. Most evidence relates to high-income Western countries [1–8], but *hi-fun* is increasingly prevalent in Southeast Asia [9–13], especially Thailand [14,15].

Although *hi-fun* is the focus of global public health concern because of links to poor mental health, sexually transmitted infections (STIs), physical and social harms [11,16], research on this topic in Thailand is at an early stage. A recent ethnography of sexualised methamphetamine use in Bangkok found MSM engaged in *hi-fun* because of associations with exclusivity, glamour, pleasure and social status [14]. Social hierarchies unique to methamphetamine parties, and the legislative environment (which harshly criminalises drug use), shape harms by contributing to a risk environment often characterised by inconsistent protective behaviours, transactional and non-consensual sex [14]. Further evidence from Thailand suggests a syndemic effect: drug use, mental health challenges, forced sex and isolation are associated with cumulative increases in MSM HIV incidence [17].

Despite a range of physical, sexual, social and mental health harms associated with *hi-fun*, comparatively little is known globally, and especially in Southeast Asia, about the specific circumstances in which individuals present to health or community services, including those harms considered most salient or impactful. In addition, there is little published research on the barriers and facilitators to implementing responses that health or community-based organisations can, or would like to, make [14]. Key informants working in policy, development, clinical and community-based organisations are especially well placed to respond to these urgent questions, thereby shaping further research priorities in this important area of focus.

This study therefore **aims** to develop an understanding of key informants’ perspectives on *hi-fun* contexts, harms and current responses in Bangkok. Our **objectives** are to 1) explore the social and cultural context of *hi-fun* in Thailand and perspectives on how and where it is practised; 2) understand the range of harms identified by key informants through their personal and professional experiences and 3) characterise barriers and facilitators to SDU service development and describe how supportive approaches have been developed.

## Methods

### Ethics statement

Ethical approval for this research was granted by Mahidol University [ref: 2020/254] and the London School of Hygiene and Tropical Medicine [ref: 22861]. All participants provided verbal recorded consent in line with Mahidol University policies.

### Study design and setting

In order to inform future research and gather a broad range of perspectives, we conducted a qualitative scoping study exploring key informants’ perspectives on the range of forces shaping *hi-fun*, as well as current provider responses.

Fieldwork occurred in Bangkok, Thailand between January and April 2021. Bangkok was selected as it is the social and economic hub of the country, and because of its status as the most important centre for gay life in Southeast Asia [14,18,19].

### Study population

Eligible participants worked in policy, development, clinical and community-based organisations serving MSM. We aimed to recruit a cross-section of professionals working in a variety of organisations targeting different segments of the MSM population, and to include staff from a range of roles (e.g. peer-workers, clinicians, managers).

### Recruitment

Key informants were recruited through the personal and professional networks of the authors. In line with scoping research approaches (which can include smaller numbers of participants) and because of budgetary considerations, we initially sought a sample size of 12 key informants from a range of organisations. Ultimately 13 were included, spread across policy, development, clinical and community-based organisations. Given that this research sought to identify a broad range complimentary and divergent avenues for further research with multiple stakeholders, we did not attempt to reach thematic saturation.

### Study procedures

A topic guide was developed by the study team and used to guide interviews. This covered contextual factors relevant to *hi-fun* (e.g. observed changes in sexual behaviour, technology and drug use), harms identified during service provision as well as barriers and facilitators to service development and implementation. Some discussed their own experiences of *hi-fun* and harms they had observed in their personal lives.

Interviews were conducted by the first and second author in English (n = 7) and Thai (n = 3) respectively and lasted between 45 minutes and 1.5 hours. Nine interviews were online (conducted through Zoom), and 1 interview was in-person. Three participants requested a group interview which was in both languages, conducted in-person and attended by the first, second and fourth author.

### Data analysis

All interviews were transcribed verbatim and Thai transcripts were translated into English. Transcripts were checked by the interviewers for accuracy. Analysis fused thematic and framework approaches: themes were identified through reading the transcripts and comparison with extant data, these were organised hierarchically, piloted on two interview transcripts (one English and one Thai) and then refined. Analysis was conducted by the first author with input from the study team. QSR Nvivo 12 was used for data organisation.

## Results

Thirteen key informants participated in interviews, four of whom reported prior *hi-fun* engagement. Table 1 provides sample details. Analysis focused on three meta-themes: *hi-fun* cultures and environments; understanding *hi-fun* harms and supportive contexts, assets and challenges.

**Table 1:** Key informant organisation types.

Organisation type	Number of key informants
Clinical	2
Community-based	7
Development	2
Policy	2

### *Hi-fun* cultures and environments

Clinical and community responses to *hi-fun* need to understand and acknowledge the practice in its social and cultural context. This first meta-theme explores the unique cultures and environments which shape *hi-fun* practices and experiences. Three primary themes were identified in analysis: technology use, innovation and norms; cultures of power and influence and drugs, tension and change.

### Technology use, innovation and norms

This theme focuses on the faciliatory role of technology in hi-fun organisation, exploring the role of both ‘open’ (e.g. Twitter, Facebook) and ‘closed’ (e.g. instant messaging groups) online spaces.

Key informants felt *hi-fun* was increasingly visible in online spaces frequented by MSM; their perception was that this SDU is primarily organised online. However, in comparison with high-income Western settings, in Bangkok this included not just geo-location social networking applications (apps) catering to MSM, but also public facing social media such as Twitter and closed discussion groups on generic online messaging applications (e.g. LINE, WhatsApp).

In addition to facilitating organisation, some participants expressed a belief that these sites normalised SDU practices, thereby providing an entry point to *hi-fun* for uninitiated MSM. This was felt especially the case through Twitter, where content frequently appeared alongside other MSM sexual material.

*I think potentially*, *it also plays some role*, *increasingly a role and maybe normalising those behaviours*, *as you know*, *part of what happens sort of in the gay scene or among MSM communities*, *just by making it somewhat*, *and I’m stressing these somewhat*, *but I do think there’s some degree on certain platforms making it somewhat more visible as well*. (Key informant, development organisation)

Indeed, several key informants discussed the increasing role of ‘influencers’ in disseminating norms around drug use online. These included individuals uploading videos of injecting methamphetamine and combining different drugs, often in highly stylised settings. However, the online context was not limited to sexualised imagery and discussion but also featured the sharing of information about harm reduction. This included advice on ways to inject safely and how to find SDU competent healthcare providers.

*So social media is used to source the drugs to connect people who want to use it*, *and also to provide information on* […] *benefits*, *and also harms*, *and ways to engage with … or to engage with the health sector and how to use chemsex more safely*. (Key informant, policy organisation)

### Cultures of power and influence

Key informants identified strong links between hi-fun and perceptions of high socioeconomic status. These associations were also seen to contribute to complex power dynamics between those with greater and lesser financial means and/or social capital.

*Hi-fun* was firmly linked to Westernisation and wealth; key informants identified that MSM engaged in *hi-fun* typically understood their drug use as ‘high-class’ in relation to other drugs which they stereotypically associated with low socioeconomic status.

***Key informant***: […] *at that time people still understood that being in this circle shows good taste*, *the sense of belonging to the high-class society*, *looking sumptuous and wealthy*.

**Interviewer**: It is the sense of belonging to the middle class or stuff like that, right?

***Key informant***: *They are so Westernized*. […] *They differentiate themselves from drug users in general like glue sniffing*, *heroin users or stuff like that*. (Key informant, clinical organisation)

Given the financial resources required to engage in *hi-fun*, and the impact of wider group dynamics, some key informants described power imbalances affecting those with less resources and/or less cultural capital. Those impacted were typically (but not exclusively) younger MSM, who often had less power and autonomy in SDU settings.

*Coercive in the sense somebody holding a gun to their head*? *No*. *But there’s financial coercion*. *There’s emotional coercion*. *There’s a desire to be part of the group that’s coercive*. *And then if you’re unconscious*, *if you are not functioning intellectually because you’re completely overwhelmed with one or another substance*, *that’s…what could you call that*? *Substance coercion*? (Key informant, development organisation)

This key informant linked imbalances in financial and cultural capital in *hi-fun* settings with uneven power dynamics leading to specific harms, including potential increases in reports of sexual assault.

### Drugs, tension and change

This theme explores the types of drugs and administration modes most closely linked with hi-fun in Bangkok, focusing on tensions between accounts of key informants.

Methamphetamine was the drug most commonly linked with *hi-fun*. However, there was substantial disagreement amongst key informants about the boundaries between *hi-fun* drugs and differing administration modes.

Several key informants felt that the introductory route to *hi-fun* in Bangkok was primarily through smoking methamphetamine. This was in comparison with those who felt that *hi-fun* in Bangkok almost exclusively involved injecting methamphetamine.

*Hi-fun is embodied by many factors*. *What I see clearest is the way they use drugs by dissolving substances in water and then injecting it into their body*. *Injection is something that*…*other types of drugs cannot be compared to because the substances will go directly to the heart of the users*. *It just makes you feel aroused completely*. *Other types of drugs cannot give the same effect*. (Key informant, community-based organisation)

A further group took a wider view on drugs associated, and linked polydrug use including MDMA, novel psychoactive substances and ketamine with *hi-fun*. This suggests a contested landscape where *hi-fun* was viewed and defined differently by various key informants.

There was substantial agreement, however, that injection of methamphetamine was common, likely more so than in other contexts. In comparison, GHB/GBL was felt to be used only by a minority. There is, as yet, insufficient population-level data pertaining to these practices to provide clarity as to the relative frequency of each, or the boundaries between *hi-fun* and other types of SDU in this context.

### Understanding *hi-fun* harms

This meta-theme details the most pressing harms identified by key informants. Harms clustered in three main areas: police, coercion and enforcement; acquisition and transmission risks and emergencies, overdose and psychosis.

### Police, coercion and enforcement

The police, and the legislative environment more broadly, were linked to harm for MSM involved hi-fun. This was the most pressing concern for key informants who themselves were engaged in hi-fun, but was identified nearly universally as a significant issue.

Key informants described potential police entrapment taking place through a number of means. Most commonly, police officers would use apps, pose as someone hosting a *hi-fun* party and lure men before fining or potentially prosecuting them for drug use.

*A friend of mine* [was] *caught by the police*, *being entrapped first*… *we tried to find out where he was and luckily a cellmate at the police centre had a mobile phone*. *So he remember my phone number*, *so he just texted me*: *“*I’m here, pick me up.*” Luckily*, *if not*, *then I’ve probably lost this my friend*. (Key informant, community-based organisation)

Some key informants reported hearing that police coerced men into luring their sexual contacts to parties, before extorting them for financial gain. This contributed to trust issues between men engaged in *hi-fun* men because of the potential for someone to be ‘turned’ by the police and begin acting as an informant.

*What can happen is that one*, *who finished before the others could leave the party and then become a spy for the police to arrest them*. *Therefore*, *safety is what they are concerned with the most* (Key informant, community-based organisation)

### Acquisition and transmission risks

There was substantial concern among key informants on the potential of SDU to lead to increases in STIs, HIV and hepatitis C (HCV).

As in many settings, most concern focused on the potential for increases in HIV and HCV acquisitions linked to *hi-fun* because of potentially low condom use and high partner numbers in these environments. This was further compounded by the sense that significant proportions inject methamphetamine, leading to increased opportunity for transmission of bloodborne viruses.

*Their main problem is related to the use of sterile needles*. […] *They usually become careless when they are emotionally carried away during sex*. *Some MSM may also carry other diseases passed through drug injections like Hepatitis C*. *At the end of the day*, *they end up using no condom and violence* [having rough sex], *which puts them at risk of blood-borne diseases anyway*. (Key informant, community-based organisation)

While increasing provision of HIV pre-exposure prophylaxis (PrEP) was felt to lessen the risks of HIV transmission, concerns around bacterial STIs were substantial. Indeed, many key informants identified that Thailand specifically did not have a sexual healthcare system that could respond to the range of STIs faced by MSM engaged in *hi-fun*. This was primarily because of resource constraints around expensive diagnostic tests.

[…] *it’s maybe a very usual concern*, *but still the hepatitis C and HIV and syphilis*, *gonorrhoea* [and] *chlamydia*. *These are things that in a country like Thailand*, *where you don’t have a very good sexual health system*, *you don’t have that luxury of doing asymptomatic gonorrhoea and chlamydia screening*. […] *When people say that*, *okay*, *I go to sexual health clinic regularly*, *what they will receive as services will only include HIV and syphilis testing because that’s cheap*, *but not for other STIs*. (Key informant, clinical organisation)

The risk from STIs was felt by key informants to affect most MSM engaged in *hi-fun*, as condom use in these settings was perceived to be inconsistent.

### Emergencies, overdose and psychosis

This theme explores the most acute, physical and mental health harms faced by MSM engaged in SDU, notably the potential for overdoses and psychosis from extended methamphetamine use.

Key informants who identified polydrug use as being part of *hi-fun* were more likely to describe concerns about deaths resulting from overdose. This was felt to be especially pressing amongst those who injected mixed substances as part of their SDU practices.

*I think it is about overdose*. *These days it seems there are several types of substances coming up*. *Some of them look so alike that we don’t know how to identify them*. *Sometimes people just mix several substances together and then inject it into their body*. *Some substances might have sedative effects*, *which could cause users to have severe reactions and lead them to have overdose symptoms*. (Key informant, clinical organisation)

Indeed, some key informants felt that a lack of understanding of the range of drugs being used in *hi-fun* settings made providing harm reduction advice challenging.

Alongside general concerns about depression and anxiety being exacerbated by stimulant drugs, several key informants identified psychotic episodes caused by extended methamphetamine use without breaks for sleep.

*If you don’t sleep two nights or three nights*, *sometimes you’re not concentrate with the orientation*. *So you hear something or you see something* [that’s not real]. *That’s because of you not sleeping enough*. *And sometime if drug user didn’t know that is beginning of acute paranoia*, *they’re not aware; they still keep continuing using* [methamphetamine], *so that means they make your mental health worse*, *or from the acute paranoia*, *maybe will become the chronic paranoia*, […] *related to the psychosis*. (Key informant, community-based organisation)

Some key informants felt that episodes of psychosis had the potential to lead to vulnerability to physical harm, or enforcement action by the police.

It was noted that, for the majority, these types of harms were uncommon and that when they occurred, they were often among further marginalised MSM and reinforced by pre-existing vulnerabilities.

*I know people who engage and enjoy chemsex on a regular or occasional basis*. *And I know people who have struggled with it for mental health reasons*, *but also people who can take it*, *leave it*, *depends*. *And so*, *I really do think that there is very individual*, *there is no blanket solution*. […] *I do think that there tends to be an intersection*, *obviously*, *which is similar to other issues with dependency and addiction around compounding vulnerabilities*. (Key informant, policy organisation)

### Supportive contexts, assets and challenges

This meta-theme explores the broader political, social and organisational contexts that help and hinder MSM SDU service development and delivery. These include the themes politics, macro-economics and wider culture; organisational contexts and cultures and approaches, aims and philosophy.

### Politics, macro-economics and wider culture

This theme relates to the political, economic and cultural considerations in hi-fun and other SDU service delivery. Specifically, we explore the role of political economy in the distribution of development funding and its impact on service provision for MSM in Bangkok. Following this, we outline cultural considerations in developing hi-fun/SDU support.

It was recognised that an important source of funding for sexual health programming was from overseas development aid, largely from the United States. Funding from these sources was often focused solely on HIV prevention or treatment and could not be used for broader services. When funding was less restrictive, substantial barriers existed surrounding *what* harm reduction services or messages could be provided.

[…] *most of my work is funded by the US government*. *And there are regulatory barriers around harm reduction*, *when you’re funded by the US government*, *what services you can provide*, *what types of language that you can use*. (Key informant, development organisation)

These barriers meant that some approaches which were valued by MSM engaged in *hi-fun* were not acceptable to funders. This was compounded by a sense of reluctance on the national level in Thailand for stakeholders to engage with issues of *hi-fun* because of multiple stigmas surrounding homosexuality, condomless sex and drug use.

*When you’re talking about key populations* […] *and then adding in a complexity of condomless sex that involves drug use*, *it is difficult for some stakeholders*, *even where they want to be more progressive*. *I think there is still a reluctance or a moral struggle there*, *but I do think that there are increasing conversations about it*, [and] *recognition of the fact that there is a higher risk*, […] *I think that those arguments can be made as a public health issue*. (Key informant, policy organisation)

Negative attitudes towards drug use within wider Thai culture were frequently cited as a further barrier to developing comprehensive services. This was most pronounced if said services were targeted towards younger MSM because of cultural imperatives against discussing sex and/or drugs with this group. Despite these barriers, MSM culture was felt to be faciliatory, with men engaged in *hi-fun* keen to engage in service development activities:

*We don’t have to use drugs ourselves to obtain information about it*. *We can learn from them* [MSM]. *We give them knowledge and information about health and they share with us their experience*. *So this is like a mutual benefit*. *They are pleased to share their information and volunteer themselves when asked to provide some qualitative data for us*. (Key informant, clinical organisation)

Despite substantial wider macro-economic, political and cultural barriers, MSM *hi-fun* culture facilitates service development and engagement, especially with trusted organisations. This underscores the fact that despite widespread cultural and political barriers, there are highly motivated MSM engaged in *hi-fun* who wish to shape service provision.

### Organisational contexts and cultures

This theme describes the organisational contexts relevant when considering support strategies for hi-fun engaged MSM in Bangkok. We explore the willingness of organisations to adapt and support MSM engaged in hi-fun, as well as barriers surrounding provider stigma towards this type of SDU.

Most organisations began working on issues related to *hi-fun* incidentally; SDU became an organisational concern through individuals presenting to services with acute needs. For some, there was a feeling that *hi-fun* was not within their purview, and they should only focus on areas directly related to their organisational objectives.

*As to what is to be done* […] *is in some ways controversial within our own organisation where there is a* [reluctance]. *We should only be interested in chemsex or any such thing only in the context of HIV transmission and that is best dealt with by the promotion of PrEP and we shouldn’t be doing anything more*. *So that is one thought in many sections within our organisation*. […] *I don’t subscribe to that thought*, *because to me the people we work with are important beyond just the dimension of HIV transmission*. (Key informant, policy organisation)

Although key informants were very keen to engage with issues surrounding SDU and a series of regional meetings had been held to explore emerging issues, several felt there was reluctance from the sexual health sector to make *hi-fun* a visible issue because it did not align with donor priorities. This was further compounded because of stigma from some service providers towards those who use drugs. It was also felt that explicitly providing *hi-fun* support may also act as a barrier for MSM in accessing services because of stigma related to drug use. Promoting or delivering services online only was a way to circumvent issues of visibility but could, of course, complicate awareness and accessibility of services.

*No one at this time wants to make it visible*, *in which I think it’s a major barrier because even though it may not be visible physically*, *it should be made visible online*, *or virtually*. […] *know that many*, *not many*, *a few*, *advocates online has been trying to do this*, *but it’s really hard to get someone to support them technically*, *as well as to link those online support to offline support*. (Key informant, clinical organisation)

It was felt to be important that skills were developed within the sector to improve the success of these initiatives, and that organisations responding to *hi-fun* sometimes lacked capacity in this area. This also includes addressing stigma towards people who use drugs, which was often dealt with effectively by collaborating with drug use organisations to providing enhanced training around the needs and experiences of MSM engaged in *hi-fun*.

### Approaches, aims and philosophy

This theme explores the approaches to support provided by organisations, as well as the underlying philosophy of their interventions.

Support for MSM engaged in *hi-fun* was often, but not always, informally developed in response to the needs that emerged during routine provision of other services. In many cases, this involved using peers to deliver support in response to individual need. Organisations also relied on collaborative working, referring MSM with specific issues arising from *hi-fun* to the services of collaborators.

*We definitely can help them if they straightforwardly express that they really need help from us*. […] *We have* [drug use organisation], *who can provide support and guidelines for participating in the rehab process*. *In case of HIV and STIs treatment*, *we also have connections with some healthcare establishments*, *where we can forward them to for the treatment*. *For those who call us for help concerning their hallucination problems* [psychosis], *we will get in touch with them to provide care until they recover*. (Key informant, community-based organisation)

Service providers frequently used peers when delivering HIV, HCV and STI specific services, both in clinical and community-based settings. These sometimes also included additional psychosocial interventions such as motivational interviewing aimed at supporting behaviour change.

*Probably the most targeted intervention we have* […] *is one of our partner agencies here in Bangkok*, *has trained our counsellors* […] *on the use of this WHO assessment and counselling support tool*, *that is specifically around substance use behaviours*. […] *If clients are coming in and reporting substance use*, *it’s around helping them to evaluate that substance use and determine whether or not it might be problematic*. *And then to work with the client on a brief cognitive intervention*, *brief motivational interviewing*, *to help them identify change goals that they might want to think about*. (Key informant, clinical organisation)

Services were usually client centred and focused on supporting people in meeting their own goals and aspirations. Delivering them by peers and/or in community settings increased intervention acceptability, relevance and impact.

Harm reduction was provided in two primary forms. Firstly, some organisations engaged with MSM online, either on Twitter or in closed messaging groups, and provided harm reduction advice. Secondly, organisations also provided physical materials to support risk reduction, including condoms, lubricant and (sometimes) safe injection packs. These initiatives, rather than being informally developed, were usually adapted from approaches observed in high-income settings.

[In] *the pack I’m just sending out the information we have say for* hi-fun *use*. *If you’re using ice or crystal meth*, *how do you do the orders* [of drug use], *how decided you have to stop*, *where you’re going to go to stop and how it feels like*, *what does it look like*, *where does it come from*, *the detailed information that we translate from a colleague* [redacted], *other organisations that have been successful*. *Not a very close* [translation], *but using the information as material*. (Key informant, community-based organisation)

While some appreciated easy access to injection equipment, distributing syringes was sometimes contentious; key informants reported that a substantial proportion of MSM felt that it might encourage drug use.

## Discussion

In this qualitative scoping study of interviews with 13 key informants working in policy, development, clinical and community-based organisations supporting the sexual health and wellbeing of MSM in Bangkok, we found Thai *hi-fun* cultures are distinct from chemsex in many other settings. Although *hi-fun* was predominantly organised online (as in other settings), in contrast to many contexts [20–22], *hi-fun* norms are disseminated and reinforced on social media and in closed discussion groups. *Hi-fun* was firmly associated with Westernised, middle-class MSM with potentially unique power dynamics emerging from asymmetries in financial and cultural capital.

As in several other settings [9,13,23–25], methamphetamine was felt to be the most important drug used in *hi-fun*. Tension existed, however, related to the range of drugs associated with *hi-fun* with some key informants identifying only methamphetamine use, while others felt poly-drug use was often included. Indeed, the boundaries between *hi-fun* and other types of SDU were not well defined within service provider accounts. This tension is present across settings in Asia [26,27]; developing locally grounded understandings of the differences between *hi-fun*/chemsex relative to other SDU is an urgent priority necessary to support intervention design.

The legislative environment shaped harms in distinct ways, with criminalisation and police violence raised as an issue by many key informants. STIs were a major concern, as were impacts on mental health and the potential for overdoses.

The political and cultural context produced specific barriers around developing holistic services for MSM engaged in *hi-fun*, especially limits on development funds. While many organisations had faciliatory cultures, drug use stigma from service providers remained a key barrier. Interventions were primarily informally developed, and services tended to harness collaborative working across the sector in meeting needs. Harm reduction advice was often adapted from other settings, mostly from Europe.

### Implications and comparisons to other research

Our scoping study confirms earlier research in Thailand exploring the role of Twitter in *hi-fun* organisation and in developing norms [14]. Although apps are central in chemsex organisation in other settings, and user generated pornography in establishing norms [22], the centrality of Twitter (an open social media platform) and of closed discussion groups appears to be unique to Thailand, or possibly the region more broadly [28]. This remains under-theorised.

Bangkok is characterised by stark wealth inequality. Indeed, Thailand is among the most unequal countries in the world [29]. Wealth inequalities have impacts on many facets of life, including in *hi-fun* organisation where unique power dynamics seem to emerge based on imbalances in financial and cultural capital. The impacts of these dynamics on the lives of MSM engaged in *hi-fun* are largely unknown, however, and require further attention.

Key informants in our sample felt injecting methamphetamine was very common, this is in comparison to a study in Malaysia which showed smoking was a more common route of administration [10]. In addition, the risks from extended methamphetamine use is an issue of concern noted in other contexts [30–32]. Further research is required to establish the patterns of drug consumption, their administration modes and impacts to prioritise effective supportive strategies.

Harms related to police entrapment and coercion as well as transmission of HIV, HCV and STIs were felt likely to be distributed evenly throughout the *hi-fun* engaged population; their frequency and severity of impact were unclear, however. While potential for HIV/HCV transmission through sharing injecting equipment during SDU was raised, research in other settings does not show this to be a substantial issue, at least in part due to the supportive role more experienced MSM provide in teaching others safe injection techniques [5,33]. However, MSM engaging in *hi-fun* and other SDU have pronounced utility for innovative sexual health interventions including new prevention technologies for HIV prevention, such as HIV self-testing and PrEP [34–36]. Given some service providers in Thailand are reluctant to engage with people who use drugs, and that peer-delivered interventions were highly acceptable, key-population led models of care should be central in supporting this group [37–39]. Research on the values and preferences of MSM engaged in *hi-fun* and other SDU is required to inform peer-led service design and delivery.

Harms related to overdose and impacts on mental health were felt to impact more marginalised MSM with greater frequency, and, potentially, severity. This remains under-theorised and evidence from Thailand, and other settings, is lacking.

The global political economy of development funding and the centrality of the United States as a donor in Thailand provides a substantial barrier to developing holistic services for MSM engaged in *hi-fun*. These issues are not unique to Thailand, and the form funding takes has long shaped the priorities of the HIV response [40–42]. This research, however, provides yet another example that urgent action is required to re-shape funding remits so the health and well-being needs of MSM, which extend far beyond HIV, can be addressed by services.

Finally, despite substantial political, economic and social barriers, organisations were successful in developing services which respond to many of the needs of MSM engaged in *hi-fun*. Because these were largely informally developed or adapted from high-income settings, developing a theoretical basis for further interventions is a priority.

### Strengths and limitations

This study provides a thorough overview of key *hi-fun* domains in Bangkok relevant for further research and service provision. Never-the-less some limitations are noted. Consistent with scoping research, our sample size is relatively modest. To counter concerns, we aimed to recruit as diverse a sample as possible. Unfortunately, we were unable to recruit representatives from organisations supporting MSM sex workers who will have distinct experiences of transactional SDU. Future research should prioritise including this group.

All key informants worked in services which address the health needs of MSM in Bangkok. As such it is likely that accounts will overestimate the severity of harms experienced as these individuals will have more contact with those experiencing the worst outcomes. Countering this, our sample includes key informants who were or had been engaged in *hi-fun* themselves, adding richness and nuance to our data. This was not the result of focussed recruitment effort but rather is a reflection of how peers often form a valued part of the workforce in organisations that serve MSM, further underlining the value of peer-led programs [43].

## Conclusion

Our research illustrates the unique social and cultural contexts of *hi-fun* in Bangkok. In particular, the boundaries between *hi-fun* and other SDU are not well defined and require further investigation. *Hi*-*fun* is firmly associated with Westernised, middle-class MSM, with unique power dynamics emerging from imbalances in social and financial capital. Criminalisation was identified as a key harm; the impact of police violence and potential strategies to support MSM experiencing it requires further urgent research. Finally, despite barriers from within and outside organisations, including constraints on development funding, key informants were able to develop and implement highly acceptable responses supporting MSM engaged in *hi-fun*. Because many of these were informally developed or adapted from high-income settings, establishing a theoretical basis for further interventions that is grounded in this unique context is a priority.
